# Fertility in male patients with familial Mediterranean fever and paternal effect of FMF on pregnancy outcomes and complications

**DOI:** 10.1007/s11739-025-03881-y

**Published:** 2025-02-05

**Authors:** Kerem Parlar, Feyza Nur Azman, Sena Ladin Sıcakyüz, Melike Rızaoğlu, Enes Azman, Mebrure Burçak Yüzbaşıoğlu, Dilvin Korkmaz, Serdal Uğurlu

**Affiliations:** 1 Cerrahpasa Medical Faculty, Istanbul University-Cerrahpasa, Istanbul, Turkey; 2 Acibadem University School of Medicine, Istanbul, Turkey; 3 Department of Internal Medicine, Cerrahpasa Medical Faculty, Istanbul University-Cerrahpasa, Istanbul, Turkey; 4 Division of Rheumatology, Department of Internal Medicine, Cerrahpasa Medical Faculty, Istanbul University-Cerrahpasa, 53 Kocamustafapasa Street, Fatih, Istanbul, Turkey 34098

**Keywords:** Familial Mediterranean fever, Colchicine, Fertility, Pregnancy, Paternal effect

## Abstract

**Objectives:**

This study investigates the impact of Familial Mediterranean Fever (FMF) and its treatment on male infertility, and the paternal effect of FMF on pregnancy outcomes or complications.

**Methods:**

We enrolled 282 adult male FMF patients and excluded 102 for never attempting pregnancy. Demographic and clinical data, including MEFV mutation status and treatment history, were collected. Fertility status and pregnancy outcomes were assessed through interviews and medical records. Statistical analysis was performed using Fisher’s exact test, with significance set at p < 0.05.

**Results:**

Among the 180 patients who attempted pregnancy, 177 (98.3%) achieved pregnancy. Only 3 (1.7%) were infertile. A total of 452 pregnancies were conceived, with 85.0% resulting in live births. Miscarriages occurred in 11.9%, stillbirths in 0.9%, and ectopic pregnancies in 0.9%. The most common complication was preterm birth (4.2%), followed by gestational diabetes (2.1%). Eleven patients with amyloidosis achieved 39 pregnancies, with no cases of infertility.

**Conclusion:**

FMF does not lead to decreased male fertility or adverse pregnancy outcomes. Colchicine is safe for use during conception. Male FMF patients and their partners do not need additional precautions during pregnancy attempts and follow-up.

## Highlights

- FMF does not decrease male fertility.
- Colchicine is safe for conception attempts.
- FMF has no paternal effect on pregnancy outcomes.

## Introduction

Familial Mediterranean Fever (FMF) is an autoinflammatory disease characterized by recurrent attacks of fever and polyserositis. These attacks are generally self-limited, typically lasting 1–7 days. Amyloidosis is the most feared complication of FMF. Most patients have a mutation in the Mediterranean fever (MEFV) gene, which is located on the short arm of chromosome 16. Colchicine is the primary treatment for this disease. In cases where patients do not respond to colchicine, anti-IL-1 therapies are used [1, 2]. The most commonly used anti-IL-1 agents are anakinra and canakinumab [3].

Several studies have shown that fertility was reduced in patients with FMF compared to healthy populations, particularly before the routine use of colchicine. More recent studies indicate that the infertility rate among FMF patients is similar to that of the general population [4, 5]. Female patients diagnosed with FMF have been reported to experience ovulatory disorders and intra-abdominal adhesions. Male patients, on the other hand, exhibit oligospermia and azoospermia [6]. A rare symptom of the disease is scrotal edema and acute orchitis, caused by inflammation of the tunica vaginalis, which can lead to testicular damage affecting spermatogenesis [7]. Testicular amyloidosis, an infrequent complication observed more commonly in untreated or treatment-resistant cases, can cause abnormalities in semen parameters by affecting seminiferous tubules and testicular vessels [8]. Due to colchicine’s mechanism of action, concerns have been raised that its use in treatment may contribute to male infertility. However, there are still conflicting reports regarding the effect of colchicine on male fertility, with some studies reporting decreased sperm parameters and others reporting no effect [9–11].

Research on the relationship between FMF and pregnancy outcomes and complications is less definitive than that on infertility. In a meta-analysis by Hirahara et al., FMF was linked to preterm birth, fetal growth restriction, and maternal hypertensive disorders in female patients [12]. Several studies have reported the presence of other pregnancy complications such as gestational diabetes and ectopic pregnancy, according to a review by Er et al. [13]. However, to the best of our knowledge, there are no studies on the paternal effect of FMF on pregnancy outcomes or complications.

As one of the clinics with one of the largest FMF cohorts in the world, we aimed to investigate the impact of FMF and its treatment on male infertility, and the paternal effect of FMF on pregnancy outcomes or complications. Prior research has explored the connection between FMF and male fertility; however, this study is the first to investigate the impact of FMF on pregnancy outcomes and complications from a paternal perspective.

## Methods

In this study, we enrolled 282 consecutive adult male patients who presented to our outpatient tertiary center clinic between 5 December 2023 and 8 January 2024. All patients were previously diagnosed with Familial Mediterranean Fever (FMF) according to the Tel-Hashomer criteria. After enrollment, patients were contacted via telephone to conduct an interview. Then, a total of 102 patients were excluded because they had never attempted pregnancy. These patients continue their follow-up at the Cerrahpasa Medical Faculty Adult Rheumatology Outpatient Clinic, a nationwide referral center.

This study was approved by the Istanbul University-Cerrahpasa Ethical Committee (approval no: OErl6mJD). This study was conducted in accordance with the principles of the Declaration of Helsinki. Informed consent was obtained from all the participants.

Demographic characteristics, MEFV mutation status, age at diagnosis, age at disease onset, total disease duration, medication history, comorbid diseases, smoking history, alcohol use history, presence of amyloidosis, and history of renal transplantation were obtained from the medical records. Family histories of FMF and infertility were also obtained. Medications used during pregnancy attempts were recorded in patients with and without children.

Each patient underwent a comprehensive interview regarding their fertility status. Infertility was defined as the inability to conceive after one year of regular unprotected intercourse. Marital status, duration of marriage, and the number of children were recorded. Patients who experienced difficulties conceiving were questioned about the potential reasons for infertility and their spouses’ fertility status. Partners’ medical histories were also examined. Available spermiogram results were obtained. History of epididymitis and orchitis was also investigated. Patients encountering fertility challenges were asked about their use of assisted reproductive techniques (ART), such as in vitro fertilization (IVF). The success rates of these procedures have also been noted.

Patients who achieved pregnancy were further interviewed. Pregnancy results and possible complications were investigated and their frequencies were recorded. Pregnancy results included live birth, first trimester miscarriage, second trimester miscarriage, stillbirth, ectopic pregnancy, anembryonic pregnancy, and elective abortion. Possible pregnancy complications included preterm birth (delivery at < 37 weeks), gestational hypertensive disorders, fetal anomalies, fetal growth restriction (fetal weight < 10th percentile), gestational diabetes, postterm birth, macrosomia, and perinatal asphyxia.

Descriptive statistics are presented as numbers and percentages for the categorical variables. They are presented as the mean ± standard deviation for numerical variables. Statistical analysis was performed using Fisher’s exact test to compare categorical variables. Statistical significance was set at p < 0.05. All analyses were performed using open-source R software v.4.1.2, running under IDE RStudio v.2023.12.1.

## Results

A total of 180 patients (63.8%) attempted to achieve pregnancy with their partners. The demographic features of the patients who attempted pregnancy are shown in Table 1. The most commonly observed comorbidities and chronic diseases among the patients’ wives are presented in Table 1. There were no comorbidities known to commonly cause infertility other than other rheumatologic conditions in our cohort. Six patients had ankylosing spondylitis, five had rheumatoid arthritis, three had gout, and two had psoriasis. Possible causes of primary infertility among the wives of our patients included endometriosis, leiomyoma uteri, and rheumatologic conditions. One patient had endometriosis, one had leiomyoma uteri, three had rheumatoid arthritis, one had ankylosing spondylitis, and one had Behcet’s disease. There were no other relevant comorbidities or chronic diseases among the patients’ wives. Eleven patients in our cohort had renal amyloidosis and one had renal transplant.

**Table 1:** Demographic features of patients with pregnancy attempts

Patients with pregnancy attempts	n = 180
Age, years (mean ± SD)	44.9 ± 10.3
Disease duration, years (mean ± SD)	22.5 ± 10.7
Renal amyloidosis, n (%)	11 (6.1)
Renal transplant, n (%)	1 (0.01)
Family history of FMF, n (%)	131 (72.8)
Family history of infertility, n (%)	14 (7.8)
Smoking history, n (%)	97 (53.9)
Alcohol use history, n (%)	39 (21.7)
Education level, n (%)	
Elementary school	49 (27.2)
High school	67 (37.2)
University/College	53 (29.4)
Masters/Doctorate	9 (0.1)
Unspecified	2 (0.01)
Comorbidities, n (%)	86 (47.8)
Diabetes mellitus	15 (8.3)
Hypertension	15 (8.3)
Atherosclerosis	7 (3.9)
Ankylosing spondylitis	6 (3.3)
Rheumatoid arthritis	5 (2.8)
Other	38 (21.1)
Chronic disease in wife, n (%)	47 (26.1)
Thyroid disease	10 (5.6)
Hypertension	6 (3.33)
Diabetes mellitus	5 (2.8)
Rheumatoid arthritis	3 (16.7)
FMF	1 (0.01)
Other	22 (12.2)
MEFV mutation status, n (%)	
M694V homozygous	22 (12.2)
M694V heterozygous	55 (30.6)
Other	41 (22.8)
No mutation present	9 (0.1)
Not available	53 (29.4)

Only three patients (1.7%) could not conceive despite twelve months of regular unprotected sexual intercourse. Two reported that their wives had problems with fertility. One of their wives had endometriosis, and the other had leiomyoma uteri. Both had unsuccessful attempts at in vitro fertilization (IVF). One patient was taking colchicine, while the other was not on any medication during their attempts to conceive. The third patient who could not conceive used colchicine while attempting pregnancy. All three patients who could not conceive had M694V homozygous MEFV mutations. In terms of educational attainment, one of these three patients had completed only elementary school, another had finished high school, and the third had a university-level education.

Of the 177 patients who achieved conception, seven used ART to achieve pregnancy. Pregnancy outcomes, drugs used during conception, and MEFV mutations in fathers are shown in Table 2. The majority of patients were on colchicine treatment (54.9%), and nearly half did not use any drug (42.9%) while attempting pregnancy.

**Table 2:** Features of pregnancies achieved

Pregnancy	n = 452
Outcome	
Live birth, n (%)	384 (85.0)
First trimester miscarriage, n (%)	35 (7.7)
Second trimester miscarriage, n (%)	7 (1.5)
Miscarriage time unspecified, n (%)	12 (2.7)
Stillbirth, n (%)	4 (0.9)
Ectopic pregnancy, n (%)	4 (0.9)
Anembryonic pregnancy, n (%)	1 (0.2)
Abortion, n (%)	5 (1.1)
Drug used during conception	
None, n (%)	193 (42.7)
Colchicine, n (%)	248 (54.9)
Colchicine and sulfasalazine, n (%)	1 (0.2)
Colchine and anakinra, n (%)	1 (0.2)
Colchicine and canakinumab, n (%)	4 (0.9)
Sulfasalazine, n (%)	3 (0.7)
Anakinra, n (%)	1 (0.2)
Canakinumab, n (%)	1 (0.2)
MEFV mutation of father	
M694V homozygous, n (%)	43 (9.5)
M694V heterozygous, n (%)	126 (27.9)
Other, n (%)	134 (29.6)
Not available, n (%)	149 (33.0)

Seven of 177 patients used ART to achieve pregnancy. Four of these patients used it to achieve their first and only pregnancy. All four resulted in live births. One patient had an ectopic pregnancy before attempting ART. He and his partner had two miscarriages via ART before having a healthy birth via ART. Four of these five patients had abnormal spermiogram findings. The other two patients who tried ART achieved successful natural pregnancies before attempting ART, and both tried ART because their wives had early menopause. One patient had a successful pregnancy via ART and the other could not achieve pregnancy via ART.

Table 3 presents the frequency of pregnancy complications among pregnancies that resulted in live births (n = 384). The most common complication was preterm birth; however, it occurred in only 4.2% of the live births. Gestational diabetes was the second most common complication, occurring in 2.1% of the live births. The frequency of every other complication was ≤ 1.0%.

**Table 3:** Frequency of pregnancy complications

Live births	n = 384
Preterm birth, n (%)	16 (4.2)
Gestational hypertensive disorders, n (%)	4 (1.0)
Gestational diabetes, n (%)	8 (2.1)
Fetal growth restriction, n (%)	4 (1.0)
Perinatal asphyxia, n (%)	1 (0.3)
Postterm birth, n (%)	3 (0.8)
Fetal anomalies, n (%)	1 (0.3)
Macrosomia, n (%)	1 (0.3)

Pregnancy outcomes and their relationship with MEFV mutation status and treatment used during conception are shown in Table 4. There were no significant associations between pregnancy outcomes and treatment or MEFV mutation status. Pregnancy complications among live births and their relationship with the MEFV mutation status and treatment used during conception are shown in Table 5. Among live births that resulted in preterm birth, the M694V allele was significantly less likely to be present (p = 0.034) in the father. MEFV mutation status of the father was available in twelve preterm births. M694V allele was not present in nine of them. Among live births that resulted in gestational hypertensive disorders, the father was significantly more likely to have used no drugs rather than colchicine during conception (p = 0.037). In all four pregnancies that resulted in gestational hypertensive disorders, the father did not use any drugs during conception. Among live births that did not result in any complications, the father was significantly more likely to harbor the M694V allele (p = 0.017). MEFV mutation status of the father was available in 234 pregnancies with no complications. M694V allele was present in 139 (59.4%). No other significant associations were observed.

**Table 4:** Pregnancy outcomes and their relationship with MEFV mutation status and treatment used during conception

	Colchicine only	No drug	Other^a^	p	M694V allele present	M694V allele not present	p
Miscarriage, n (%)	32 (59.3)	21 (38.9)	1 (1.9)	0.836	19 (51.4)	18 (48.6)	0.563
Ectopic pregnancy, n (%)	2 (50.0)	2 (50.0)	0 (0)	1	1 (50.0)	1 (50.0)	1.000
Anembryonic pregnancy, n (%)	1 (100.0)	0 (0)	0 (0)	1	0 (0)	1 (100.0)	0.442
Stillbirth, n (%)	1 (25.0)	3 (75.0)	0 (0)	0.387	0 (0)	1 (100.0)	0.442
Live birth, n (%)	212 (54.5)	167 (42.9)	10 (2.6)	0.926	149 (56.9)	113 (43.1)	0.398

^a^(Colchicine and sulfasalazine) or (colchicine and anakinra) or (colchicine and canakinumab) or sulfasalazine or anakinra or canakinumab

**Table 5:** Pregnancy complications among live births and their relationship with MEFV mutation status and treatment used during conception

	Colchicine only	No drug	p	M694V allele present	M694V allele not present^a^	p
Preterm birth, n (%)	11 (68.8)	5 (31.3)	0.291	3 (25.0)	9 (75.0)	**0.034**
Gestational hypertensive disorders, n (%)	0 (0)	4 (100.0)	**0.037**	0 (0)	2 (100.0)	0.185
Gestational diabetes, n (%)	4 (50.0)	4 (50.0)	0.735	4 (57.1)	3 (42.9)	1
Fetal growth restriction, n (%)	2 (50.0)	2 (50.0)	1	1 (25.0)	3 (75.0)	0.318
Perinatal asphyxia, n (%)	1 (100.0)	0 (0)	1	0 (0)	0 (0)	–
Postterm birth, n (%)	2 (66.6)	1 (33.3)	1	1 (100.0)	0 (0)	1
Fetal anomalies, n (%)	1 (100.0)	0 (0)	1	0 (0)	1 (100.0)	0.431
Macrosomia, n (%)	0 (0)	1 (100.0)	0.441	1 (100.0)	0 (0)	1
No complications, n (%)	191 (56.0)	150 (44.0)	0.93	139 (59.4)	95 (40.6)	**0.017**

^a^Patients with unspecified MEFV mutations were not included

Bold values are statistically significant (p < 0.05)

Eleven patients with amyloidosis achieved thirty-nine pregnancies. None of the patients were infertile. There were 30 live births (76.9%), six miscarriages (15.4%), two abortions (5.1%), and one ectopic pregnancy (2.6%). Only colchicine was used during the conception of twenty-eight pregnancies (71.8%), colchicine and canakinumab were used together during the conception of two pregnancies (5.1%). No drugs were used during the conception of nine pregnancies (23.1%). Two live births were complicated by preterm birth (6.7%) and one by gestational hypertensive disorder (3.3%).

## Discussion

In this study, we evaluated adult male FMF patients to determine their fertility status and assess whether FMF exerts a paternal effect on pregnancy outcomes or complications. There are prior studies regarding the relationship between FMF and male fertility; however, this is the first study to analyze the paternal effect of FMF on pregnancy outcomes or complications. Among the 180 patients who attempted pregnancy, 47.7% had other comorbidities. Among the 127 patients with available MEFV mutations, 77 (60.6%) had the M694V allele and 22 (17.3%) were homozygous. The mean disease duration was 22.5 ± 10.7 years and 11 patients (6.1%) had amyloidosis.

Multiple studies have reported an increase in the incidence of infertility in male patients with FMF. The most commonly proposed mechanisms include colchicine use, testicular amyloidosis, and inflammation itself [13]. In our cohort, only three patients (1.7%) reported infertility. The male infertility rate worldwide is approximated to be 10–15% [14]. The prevalence of male infertility in our cohort was considerably lower than the population average. This may be due to the higher health literacy and regular follow-up care of our patients. Due to their chronic disease, they are more attentive to fertility concerns and often consult their rheumatologists before trying to conceive. The three infertile patients in our study had different levels of education. Testicular amyloidosis is unlikely to be present in any patient in our cohort as none of the eleven patients with amyloidosis in our cohort was infertile.

Studies regarding male fertility in FMF are fewer in number, and existing studies often involve smaller sample sizes than those focusing on female patients. The study by Atas et al. [5] was relatively recent (2019) and included 199 male patients. Eight (4%) patients were infertile. In their cohort, 74.9% of patients had the M694V allele; however, this data was given for both male and female patients. Their cohort had a mutation profile similar to ours, and the male infertility rate was lower than the population average like our cohort. There were also patients with reversal of infertility when switched from colchicine to anti-IL-1 agents in their cohort. Additionally, reversal of infertility after switching to anti-IL-1 agents has been reported in case reports [15, 16]. None of the patients in our cohort switched from colchicine to anti-IL-1 agents to achieve pregnancy. This was because of the low number of infertile patients and the low number of patients who used anti-IL-1 agents in our cohort. The low incidence of anti-IL-1 agent use in our cohort is a limitation of our study.

Research on the relationship of FMF with pregnancy outcomes and complications is less definitive than that on infertility. In female patients, FMF was linked to preterm birth, fetal growth restriction, and maternal hypertensive disorders in a meta-analysis by Hirahara et al. [12]. Several studies have reported the presence of other pregnancy complications such as gestational diabetes and ectopic pregnancy, according to a review by Er et al. [13]. However, there are no prior studies on the paternal effect of FMF on pregnancy outcomes or complications. In our cohort, 85.0% of the pregnancies resulted in live births. The rate of miscarriage was 11.9%. This is lower than the Turkish population average of 13% [17]. The majority of miscarriages in our cohort were in the first trimester, similar to the general population. First-trimester miscarriages are generally not associated with any drugs or other etiologies.

The stillbirth rate was 0.9%, which is similar to the Turkish population average of 1% [17]. The ectopic pregnancy rate was 0.9%, which is lower than the global average of 2% [18]. Colchicine was used in most pregnancies, and no drug was used in nearly half of the pregnancies in our cohort. These values show that there is no paternal effect of FMF on pregnancy outcomes, such as miscarriage, stillbirth, or ectopic pregnancy. As the majority of pregnancies in our cohort were conceived during colchicine use, we can also conclude that colchicine is safe to use during conception regarding the paternal effect on pregnancy outcomes. The elective abortion rate in our cohort was 1.1%, which is lower than the Turkish population average of 6% [17]. We hypothesize that this is because FMF patients are more likely to deliberately plan before conceiving compared to the general population, as they are handling a chronic disease.

Preterm birth was the most common complication in our cohort, with a prevalence of 4.2%. This is much lower than the Turkish population average of 12.9% [19]. The rate of gestational diabetes was 2.1%, which is lower than the Turkish population average of 8.1% [20]. The frequency of every other pregnancy complication in our cohort was ≤ 1.0% (Table 3). The frequency of gestational hypertensive disorders and post-term birth in the general population was 15.9% [21] and 7% [22], respectively. These values show that there is no paternal effect of FMF on pregnancy complications and that colchicine is safe to use during pregnancy attempts. Male FMF patients should not have any concerns regarding colchicine use during conception and can use colchicine without any precautions. Only seven pregnancies in our cohort were conceived while the father was using anti-IL-1 agents. Therefore, we cannot draw any conclusions regarding the paternal effect of anti-IL-1 agents on pregnancy outcomes or complications, which is a limitation of our study.

We identified three statistically significant associations when examining pregnancy outcomes in relation to MEFV mutation status and treatment used during conception. Among live births that resulted in preterm birth, the M694V allele was significantly less likely to be present (p = 0.034) in the father. As the presence of the M694V allele is correlated with increased disease activity, this finding supports the claim that FMF does not have a paternal effect on preterm births. Among live births that resulted in gestational hypertensive disorders, the father was significantly more likely to have used no drugs rather than colchicine during conception (p = 0.037). This association may be due to suppressed local inflammation in the testicles as a result of colchicine use, which in turn leads to increased sperm quality. However, these two findings should be considered with skepticism, as the prevalence of both preterm birth and gestational hypertensive disorders was 16 and four respectively in our cohort. Low sample sizes limit our ability to draw conclusions from these findings, and studies with larger cohorts should be conducted to support these findings. Our final significant finding was that among live births that did not result in any complications, the father was significantly more likely to carry the M694V allele (p = 0.017). We hypothesize that this may be due to increased immune privilege in the testis as a response to increased systemic inflammation in patients carrying the M694V allele.

The main limitation of our study is its retrospective nature. Most of the data were collected via retrospective chart review and telephone interviews. The interview portion may have introduced recall bias. Therefore, future prospective studies are needed to validate our findings and further clarify the paternal role of FMF in pregnancy outcomes.

To conclude, this is the first study to analyze the paternal effect of FMF on pregnancy outcomes or complications. The presence of FMF did not lead to a decrease in male fertility. FMF did not have a paternal effect on pregnancy outcomes or complications. Colchicine does not have a paternal effect on pregnancy outcomes or complications, and does not cause infertility in male patients. Colchicine cessation was not necessary prior to conception. Male patients with FMF can attempt pregnancy without precautions. The partners of male patients with FMF do not need additional precautions during pregnancy follow-up.

## Data Availability

All data relevant to the study are included in the article.

## References

[1] Ozen S, Bilginer Y (2014) A clinical guide to autoinflammatory diseases: familial Mediterranean fever and next-of-kin. Nat Rev Rheumatol 10(3):135–147. 10.1038/nrrheum.2013.174 24247370 10.1038/nrrheum.2013.174

[2] Ozdogan H, Ugurlu S (2019) Familial Mediterranean fever. Presse Med 48(1 Pt 2):e61–e76. 10.1016/j.lpm.2018.08.014 30686512 10.1016/j.lpm.2018.08.014

[3] Parlar K, Ates MB, Egeli BH, Ugurlu S (2024) The clinical role of anakinra in the armamentarium against familial Mediterranean fever. Expert Rev Clin Immunol 20(5):441–453. 10.1080/1744666X.2023.2299230 38133629 10.1080/1744666X.2023.2299230

[4] Cerquaglia C, Verrecchia E, Fonnesu C et al (2010) Female reproductive dysfunction in familial Mediterranean fever patients with and without colchicine treatment. Clin Exp Rheumatol 28(4 Suppl 60):S101 20868586

[5] Atas N, Armagan B, Bodakci E et al (2020) Familial Mediterranean fever-associated infertility and underlying factors. Clin Rheumatol 39(1):255–261. 10.1007/s10067-019-04773-1 31502094 10.1007/s10067-019-04773-1

[6] Mijatovic V, Hompes PG, Wouters MG (2003) Familial Mediterranean fever and its implications for fertility and pregnancy. Eur J Obstet Gynecol Reprod Biol 108(2):171–176. 10.1016/s0301-2115(02)00417-7 12781406 10.1016/s0301-2115(02)00417-7

[7] Schuppe HC, Pilatz A, Hossain H et al (2010) Orchitis und Infertilität [Orchitis and male infertility]. Urologe A 49(5):629–635. 10.1007/s00120-010-2256-1 20449780 10.1007/s00120-010-2256-1

[8] Haimov-Kochman R, Prus D, Ben-Chetrit E (2001) Azoospermia due to testicular amyloidosis in a patient with familial Mediterranean fever. Hum Reprod 16(6):1218–1220. 10.1093/humrep/16.6.1218 11387295 10.1093/humrep/16.6.1218

[9] Ehrenfeld M, Levy M, Margalioth EJ, Eliakim M (1986) The effects of long-term colchicine therapy on male fertility in patients with familial Mediterranean fever. Andrologia 18(4):420–426. 10.1111/j.1439-0272.1986.tb01801.x 3752545 10.1111/j.1439-0272.1986.tb01801.x

[10] Haimov-Kochman R, Ben-Chetrit E (1998) The effect of colchicine treatment on sperm production and function: a review. Hum Reprod 13(2):360–362. 10.1093/humrep/13.2.360 9557838 10.1093/humrep/13.2.360

[11] Ben-Chetrit E, Backenroth R, Haimov-Kochman R, Pizov G (1998) Azoospermia in familial Mediterranean fever patients: the role of colchicine and amyloidosis. Ann Rheum Dis 57(4):259–260. 10.1136/ard.57.4.259 9709191 10.1136/ard.57.4.259 PMC1752571

[12] Hirahara Y, Yamaguchi M, Takase-Minegishi K et al (2024) Pregnancy outcomes in patients with familial Mediterranean fever: systematic review and meta-analysis. Rheumatology (Oxford) 63(2):277–284. 10.1093/rheumatology/kead417 37594755 10.1093/rheumatology/kead417

[13] Er O, Ugurlu S (2024) Fertilization, reproductive system, and pregnancy in familial Mediterranean fever: clinical state of art. Mod Rheumatol 34(2):265–271. 10.1093/mr/road067 37405693 10.1093/mr/road067

[14] Anawalt BD. Causes of male infertility. In: Connor RF (ed) UpToDate, Wolters Kluwer. Accessed on July 8, 2024

[15] Egeli B, Parlar K, Filiz B, Durucan I, Ugurlu S (2024) Novel use of interleukin-1 antagonists in male familial Mediterranean fever patients with infertility: case series. Arch Rheumatol 39(3):474–475. 10.46497/ArchRheumatol.2024.10269 39507831 10.46497/ArchRheumatol.2024.10269 PMC11537677

[16] İlgen U, Küçükşahin O (2017) Anakinra use during pregnancy: report of a case with Familial Mediterranean Fever and infertility. Eur J Rheumatol 4(1):66–67. 10.5152/eurjrheum.2017.16075 28293457 10.5152/eurjrheum.2017.16075 PMC5335891

[17] Hacettepe University Institute of Population Studies (2019) 2018 Turkey Demographic and Health Survey. Hacettepe University Institute of Population Studies, Presidency of the Republic of Turkey Strategy and Budget Office, and TÜBİTAK, Ankara, Turkey.

[18] Lee IT, Barnhart KT (2023) What is an ectopic pregnancy? JAMA 329(5):434. 10.1001/jama.2022.22941 36749331 10.1001/jama.2022.22941

[19] Public Health General Directorate. 17 Kasım Dünya Prematüre Günü. Republic of Turkey Ministry of Health. Published July 5, 2023. Accessed July 9, 2024. https://hsgm.saglik.gov.tr/tr/haberler-cocukergen/dunya-premature-gunu.html

[20] Kaya R, Karaçam Z (2019) Gestasyonel Diyabet Görülme Sıklığı ve Anne-Bebek Sağlığı ile İlişkisi. DÜ Sağlık Bil Enst Derg Ocak 9(1):10–18. 10.33631/duzcesbed.397362

[21] (2022) Hypertensive disorders in pregnancy and mortality at delivery hospitalization—United States, 2017–2019. MMWR Morb Mortal Wkly Rep 71(17):602–608. 10.15585/mmwr.mm7117a1 10.15585/mmwr.mm7117a1 PMC9098235 35482575

[22] Martin JA, Hamilton BE, Sutton PD et al (2007) Births: final data for 2005. Natl Vital Stat Rep 56(6):1–103 18277471

